# CT引导下经皮肺穿刺对于实性肺小结节的诊断：单中心经验总结

**DOI:** 10.3779/j.issn.1009-3419.2020.103.03

**Published:** 2020-06-20

**Authors:** 文峰 俞, 舟 安, 志田 王, 望 吕, 坚 胡

**Affiliations:** 310003 杭州，浙江大学医学院附属第一医院胸外科 Department of Thoracic Surgery, the First Affiliated Hospital of Zhejiang University, Zhejiang University School of Medicine, Hangzhou 310003, China

**Keywords:** 经皮肺穿刺, 肺小结节, 经验总结, Percutaneous lung puncture, Pulmonary nodules, Experience summary

## Abstract

**背景与目的:**

探究经皮肺穿刺对于实性肺小结节（直径≤15 mm）的诊断价值。

**方法:**

本研究回顾性地纳入了2014年1月-2018年12月于本中心行经皮肺穿刺的20例实性肺小结节患者，其中男性11例，女性9例。病灶最大直径介于0.5 cm-1.5 cm之间，排除严重脏器功能不全，有凝血障碍患者。

**结果:**

20例患者全部取材成功，19例患者均获得明确的病理诊断，其中11例患者找到恶性肿瘤细胞，明确为肺恶性肿瘤，5例为肺慢性炎，2例纤维组织增生，1例找到肺软骨组织，1例未见肿瘤细胞。穿刺后少量气胸1例，穿刺侧少量胸腔积液患者1例。

**结论:**

经皮肺穿刺对于实性肺小结节的诊断具有较高的有效性以及安全性。

## Abstract (en)

**Background and objective:**

To investigate the diagnostic significance of percutaneous lung puncture for solid pulmonary nodules (diameter ≤15 mm).

**Methods:**

This study retrospectively included 20 patients with solid pulmonary nodules who underwent percutaneous puncture from January 2014 to December 2018, including 11 males and 9 females. The diameter of the lesion is between 0.5 cm-1.5 cm, excluding severe organ dysfunction, and patients with coagulopathy.

**Results:**

All 20 patients were successfully selected, and 19 patients were diagnosed with pathological diagnosis. Among them, 11 patients found malignant tumor cells, which were clearly malignant tumors of the lungs, 5 cases of chronic inflammation of the lungs, 2 cases of fibrous tissue hyperplasia, and 1 case of lung cartilage tissue, no tumor cells were found in 1 case. One patient with a small amount of pneumothorax after puncture and one patient with a small amount of pleural effusion on the puncturesite.

**Conclusion:**

Percutaneous lung puncture has a high effectiveness and safety for the diagnosis of solid pulmonary nodules.

肺癌是现今癌症死亡的最主要原因。非小细胞肺癌约占肺癌的85%^[1]^。肺癌的5年生存期呈现着明显的阶梯式下降，从Ia期90%的5年生存率到IIIc期10%的5年生存率，早期和晚期肺癌预后相差巨大。早期的及时诊断对于预后有着重大意义，早期诊断、及时治疗的患者往往可以达到较满意的治疗效果^[2]^。肺癌的早期诊断依赖于影像学及病理活检两种手段，获取肺结节病理有经皮肺穿刺和经支气管镜活检两种常见方法。目前，由于胸部低剂量计算机断层扫描（low-dose computed tomography, LDCT）筛查的推广，肺小结节（≤1.5 cm）的检出率逐年升高，对于纯磨玻璃、混合磨玻璃肺结节，一般良恶性争议较小，绝大多数都能把握外科介入时机。而实性肺小结节的良恶性鉴别诊断是一个胸外科的难点。单独影像学的诊断在肺小结节的性质判定中还是具有一定的局限性。对于偏外周型肺小结节占位，支气管镜病理活检难度较大，经皮穿刺活检广泛用于偏外周肺小结节的良恶性诊断。这个诊断方法具有准确、微创的特点，通过在手术前提供较为明确的诊断和更灵活的治疗方案而使患者受益。多中心研究^[3]^显示经皮肺穿刺总体的精确度在90%以上；灵敏度为92%；特异度为86.5%；失败率接近10%。肺结节大小、所处肺叶、距胸膜距离以及患者结节是否在肺大泡周围、穿刺的时间都会影响穿刺的正确率。其中肺结节直径小于10 mm是穿刺失败肺结节诊断的独立影响因素^[4]^。直径在1.5 cm以下的肺小结节穿刺具有一定的难度，主要为穿刺定位困难，取样难以取到足够有效的标本量，其有效性和准确性以及可能出现的并发症将在本研究中进行分析。

## 1 资料与方法

### 1.1 一般资料

纳入2014年1月-2018年12月于本中心行CT引导下经皮肺穿刺活检的20例肺小结节（≤1.5 cm）的患者，其中男性11例，女性9例。病灶为肺部占位，直径介于0.5 cm-1.5 cm（10.65±2.3）mm之间，排除严重脏器功能不全、有凝血障碍患者。患者CT征象见图 1，患者一般资料见表 1。

**1:**
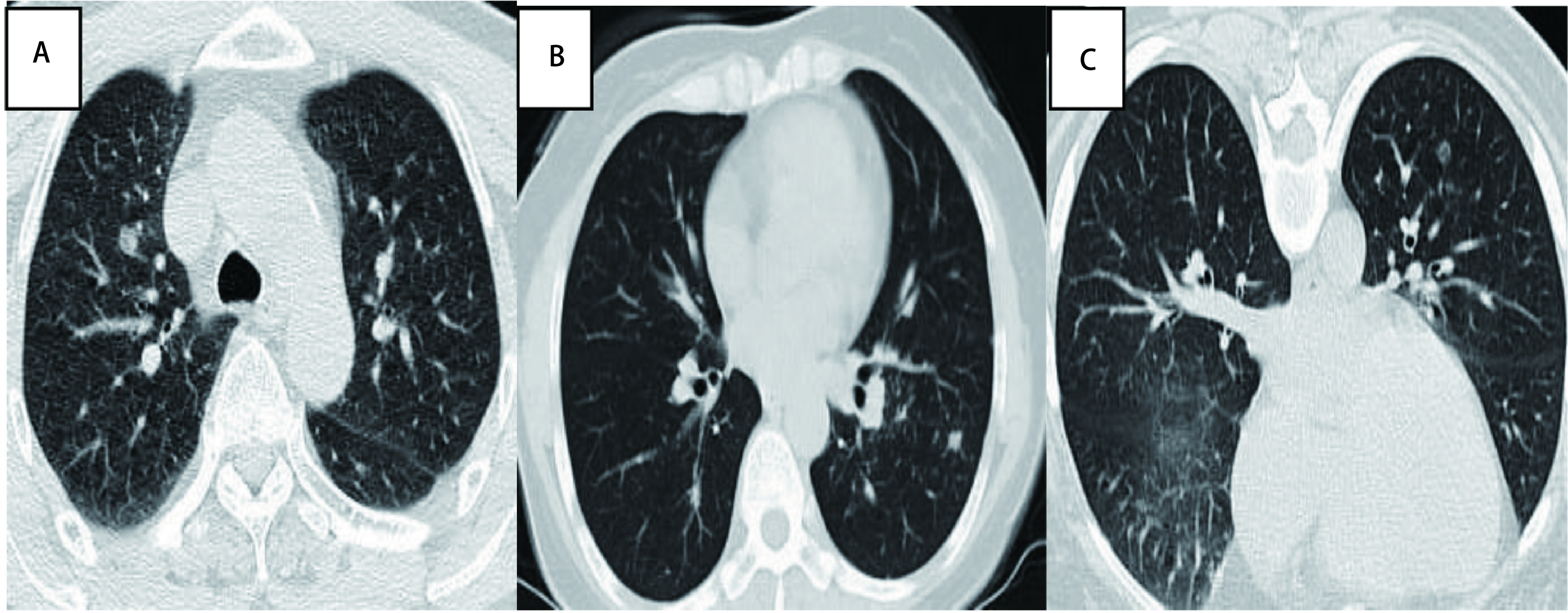
患者CT征象。A：右肺上叶实性小结节；B：左肺下叶亚厘米结节；C：左肺下叶亚厘米磨玻璃结节 CT signs of patients. A: right upper lobe solid nodules; B: left lung nodules; C: left lower lobe sub-centimeter ground-glass nodules. CT: computed tomography.

**1:** 基线数据 Baseline characteristics

Baseline characteristics	
Number of patients	20
Gender	
Male	11
Female	9
Age (yr)	57.2±10.2
Size (mm)	10.65±2.3
Position	
Left	11
Right	9
Complication	
Pneumothorax	1
Pleural effusion	1

### 1.2 治疗方法

患者术前常规行256排螺旋CT扫描，根据肺小结节的位置取合适的体位，确定穿刺位置，做出标记，确定穿刺角度，局麻，嘱患者屏气，快速进针至病灶处。CT扫描，确认针尖位置在病灶内，切割肿块活检，快速拔针，标本送检。术后再次行CT扫描，确定有无出血、气胸等并发症。术后予病房生命监护，观察无殊后予出院。

## 2 结果

结果分为以下诊断类别：找到恶性肿瘤细胞，包括具体分型、疑似恶性肿瘤和未找到恶性肿瘤细胞。找到恶性肿瘤细胞和疑似恶性可作为阳性结果。穿刺的结节的最终有2种结局：①手术切除：病理分析结果显示恶性肿瘤或疑似恶性或者隐球菌等真菌感染，这些结果被接受作为最终的诊断。②随访：如结果提示慢性炎症那么将进行持续的随访。在随访的过程中当结节直径减少了20%以上或在大小上至少维持2年的稳定可以确认结节的风险较小。本中心行经皮肺穿刺的20例患者中，19例患者都获得明确的病理诊断，其中11例患者找到恶性肿瘤细胞，明确为肺恶性肿瘤，5例为肺慢性炎症，2例纤维组织增生，1例找到肺软骨组织，1例未见肿瘤细胞。其中有3例患者接受手术治疗，术后病理进一步证实肿瘤。有6例穿刺提示非恶性肿瘤患者进行随访无进展，证实为良性病灶。穿刺后少量气胸1例，穿刺侧少量胸腔积液患者1例（图 2、图 3）。

**2:**
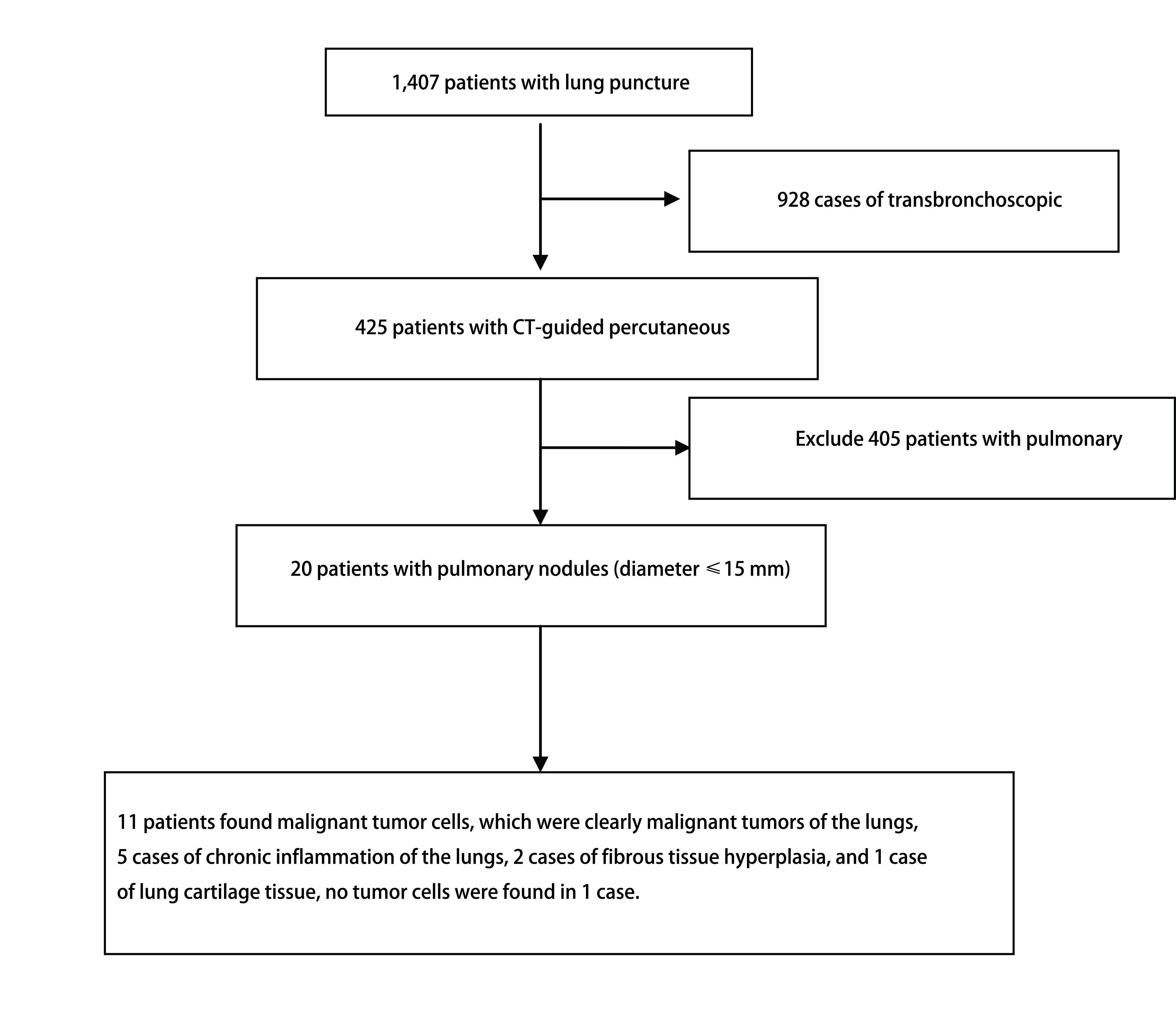
流程图示 Flow chart

**3:**
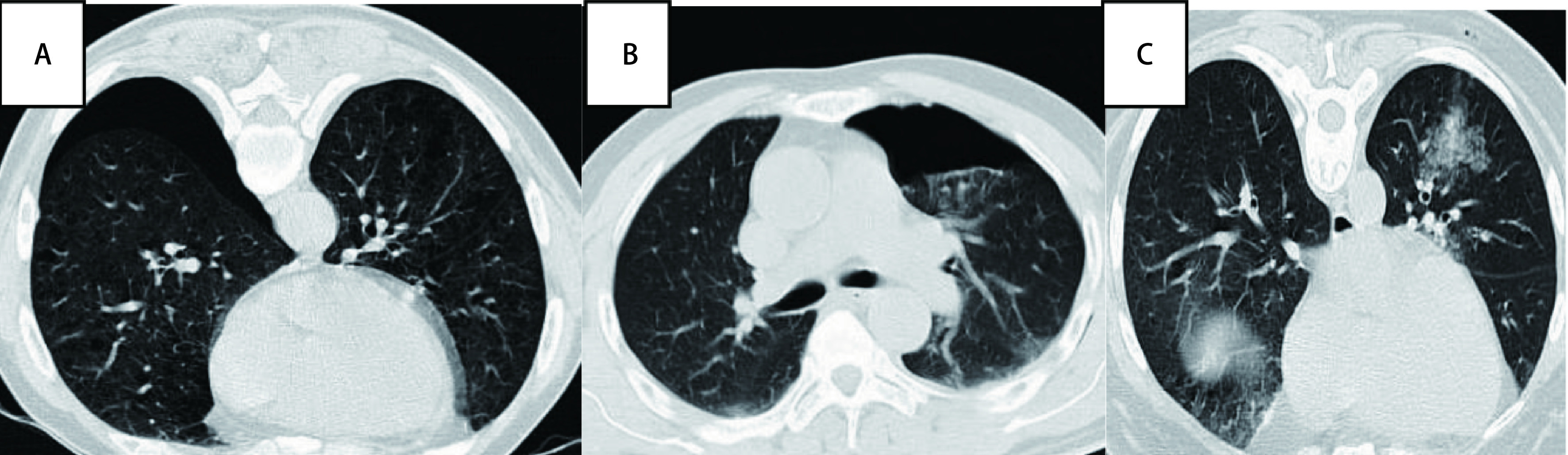
气胸和肺充血。A：气胸；B：气胸；C：肺淤血。 Pneumothorax and pulmonary congestion. A: pneumothorax; B: pneumothorax; C: pulmonary congestion.

## 3 讨论

实性肺小结节的良恶性判断是胸外科领域的诊断难点，现行的主流无创诊断技术为胸部薄层CT平扫及全身正电子发射计算机断层显像（positron emission tomography-CT, PET-CT）检查，单凭影像学诊断仍存在一定的误诊概率。当检出的肺小结节具有恶性肿瘤的影像学特征时，诊治时需要进行随访观察，当随访观察具有一定的风险或者仍是难以诊断时，如何获取病理是我们首先要考虑的。经皮穿刺肺活检、支气管镜活检和胸腔镜活检是肺部病变常用的取病理方法。支气管镜检对于肺结节的位置要求较高，而胸腔镜下活检相较于经皮肺穿刺创伤较大。所以在难以明确诊断的情况下，经皮肺穿刺活检是诊断首选^[5]^。不可否认的是侵入性活检可能导致出血、气胸以及针道转移等并发症，具有潜在的复发风险^[6]^。穿刺前后，部分患者穿刺后肺组织有淤血、胸腔积气等。

多中心研究显示气胸的发生率为25%-36%。患者年龄、性别、吸烟史、病灶的位置及大小、肺气肿及进针次数都是气胸发生的相关因素。其中病灶<2 cm、结节距胸膜超过4cm是气胸的显著影响因素。小病灶取样较为困难，会多次进针以期取到足够的标本，从而导致气胸的发生。结节临近胸膜也是气胸的影响因素，距离膈肌越近，气胸发生率越高^[7, 8]^。大部分经皮穿刺发生气胸的患者都无明显胸闷胸痛症状，只有少部分需要行胸腔闭式引流治疗^[9]^。

穿刺后咯血并不是常见的并发症，总体发生率在5%左右。女性、病灶超过2 cm、亚实性结节、深层病灶这些都是公认的咯血危险因素。女性患者咯血的发生率更高与其亚实性结节发生率高有相关性。耐人寻味的是，在一些回顾性研究中，肺气肿患者的咯血发生率反而较低。考虑可能是肺气肿患者肺组织呈退行性改变，毛细血管退化重构所致^[10]^。因此在穿刺的过程中，需要充分评估穿刺路径，避免血管切割及支气管损伤，减少咯血的发生。同时对于亚实性肺结节，穿刺的选择需慎重的斟酌。

有部分研究报道经皮肺穿刺活检可以导致针道转移及胸膜种植，这也是困扰临床医师对于是否行肺穿刺明确诊断的一个主要因素。一项回顾性研究显示，对于I期-III期肺癌患者，经皮肺穿刺明确诊断后行手术切除患者对比直接手术的患者，经皮肺穿刺活检并不会影响其死亡率。但是对临床分期I期-II期的患者来说，经皮肺穿刺有增加其远处转移的风险^[11]^。也有回顾性的研究表示经皮肺穿刺的患者对I期肺癌的患者，在手术前行经皮肺穿刺明确诊断并不会增加其复发风险，这部分患者的无病生存期（disease-free survival, DFS）并未有明显变化^[12-14]^。总的来说，对于早期的小结节患者来说，经皮肺穿刺活检是一项安全的方法，并不会导致死亡率升高。对于III期以后的患者及鳞癌患者，其预后与辅助治疗，如化疗等有相关性。但是，在我们的操作过程中，应当注意采取保护措施，极力避免针道转移及种植转移的发生。

综合多中心的研究来看，经皮肺穿刺的准确率及灵敏度都较高。失败率一般在10%左右，肺结节大小及位置、良性病变是诊断失败的独立影响因素^[15]^。其中肺结节小于10 mm极容易导致穿刺失败，结节太小，取样困难，肺叶随着呼吸摆动，细针穿刺抽吸活检难以取到足够的标本量。对于小病灶患者，可以采取经皮穿刺切割活检的方式获取标本。切割活检可以取到足够的病理标本，且并发症的发生率较细针穿刺抽吸活并没有明显的增高^[16, 17]^。经皮肺穿刺的病理结果一般分为以下几种：明确恶性；特定的良性疾病，如错构瘤等；未见肿瘤细胞；找见异型细胞。在临床实践中，穿刺病理若为找到异型细胞及不典型腺瘤样增生，结节为恶性的可能性较大。可以考虑再次穿刺明确诊断或者行手术切除病灶。对于良性病灶，则可进行密切的随访，直到排除恶性风险。

从本中心的经验来看，CT引导下穿刺实性肺小结节具有一定的可行性和诊断准确性。技术成功率较高，整体诊断准确率较高，并未发生临床恶性事件，具有较好的临床意义。近年来，C臂CT及虚拟导航也被用于肺结节的穿刺，有助于提高准确率^[18]^。本中心也在进行更多的探究。电磁导航支气管镜肺活检术及虚拟导航引导经皮肺穿刺也被广泛用于术前诊断肺小结节性质。总而言之，CT引导的经皮肺穿刺是诊断实性小肺结节的有效方法。但是，本研究纳入病例较少，没有对照，具有一定的局限性，并且为回顾性研究，需要开展相关的前瞻性研究进一步明确经皮肺穿刺对于肺小结节的诊断意义。

## References

[1] Chen W, Zheng R, Baade PD (2016). Cancer statistics in China, 2015. CA Cancer J Clin.

[2] Cheng TYD, Cramb SM, Baade PD (2017). The International Epidemiology of Lung Cancer: latest trends, disparities, and tumor characteristics. J Thorac Oncol.

[3] Lee KH, Lim KY, Suh YJ (2019). Diagnostic accuracy of percutaneous transthoracic needle lung biopsies: a multicenter study. Korean J Radiol.

[4] Tongbai T, McDermott S, Kiranantawat N (2019). Non-diagnostic CT-guided percutaneous needle biopsy of the lung: Predictive factors and final diagnoses. Korean J Radiol.

[5] Barnett J, Belsey J, Tavare AN (2019). Pre-surgical lung biopsy in management of solitary pulmonary nodules: a cost effectiveness analysis. J Med Econ.

[6] AhnJH, Jang JG (2019). Initial experience in CT-guided percutaneous transthoracic needle biopsy of lung lesions performed by a pulmonologist. J Clin Med.

[7] Patel MV, Ahmed O, Jilani D (2014). Computed tomography-guided percutaneous lung biopsy: Impact of lesion proximity to diaphragm on biopsy yield and pneumothorax rate. J Thorac Imag.

[8] Portela de Oliveira E, Souza CA, Inacio JR (2020). Imaging-guided percutaneous biopsy of nodules ≤1 cm: study of diagnostic performance and risk factors associated with biopsy failure. J Thorac Imaging.

[9] Lim WH, Park CM, Yoon SH (2018). Time-dependent analysis of incidence, risk factors and clinical significance of pneumothorax after percutaneous lung biopsy. Eur Radiol.

[10] Yoon SH, Park CM, Lee KH (2019). Analysis of complications of percutaneous transthoracic needle biopsy using CT-guidance modalities in a multicenter cohort of 10568 biopsies. Korean J Radiol.

[11] Xi Y, Fan J, Che D (2017). Distant metastasis and survival outcomes after computed tomography-guided needle biopsy in resected stage I-III non-small cell lung cancer. J Cancer.

[12] Hu C, Jiang J, Li Y (2018). Recurrence risk after preoperative biopsy in patients with resected early-stage non-small-cell lung cancer: a retrospective study. Cancer Manag Res.

[13] Ahn SY, Yoon SH, Yang BR (2019). Risk of pleural recurrence after percutaneous transthoracic needle biopsy in stage I non-small-cell lung cancer. Eur Radiol.

[14] Yang W, Sun W, Li Q (2015). Diagnostic accuracy of CT-guided transthoracic needle biopsy for solitary pulmonary nodules. PLoS One.

[15] Li GC, Fu YF, Cao W (2017). Computed tomography-guided percutaneous cutting needle biopsy for small (≤ 20 mm) lung nodules. Medicine.

[16] Kiranantawat N, McDermott S, Fintelmann FJ (2019). Clinical role, safety and diagnostic accuracy of percutaneous transthoracic needle biopsy in the evaluation of pulmonary consolidation. Respir Res.

[17] Iannelli G, Caivano R, Villonio A (2018). Percutaneous computed tomography-guided lung biopsies using a virtual navigationguidance: Our experience. Cancer Invest.

[18] Kiranantawat N, Petranović M, McDermott S (2019). Feasibility and accuracy of CT-guided percutaneous needle biopsy of cavitary pulmonary lesions. Diagn Interv Radiol.

